# Microwave Ablation Compared with Radiofrequency Ablation for The Treatment of Liver Cancer: a Systematic Review and Meta-analysis

**DOI:** 10.2478/raon-2021-0030

**Published:** 2021-06-25

**Authors:** Antonios E. Spiliotis, Gereon Gäbelein, Sebastian Holländer, Philipp-Robert Scherber, Matthias Glanemann, Bijendra Patel

**Affiliations:** 1 Department of General, Visceral, Vascular and Pediatric Surgery, University Clinic of Saarland, Homburg, Saarland, Germany; 2 Barts Cancer Institute, Queen Mary University of London, London, UK

**Keywords:** liver, carcinoma hepatocellular, liver neoplasms, radiofrequency ablation, microwaves

## Abstract

**Background:**

Guidelines have reported that although microwave ablation (MWA) has potential advantages over radiofrequency ablation (RFA), superiority in efficacy and safety remain unclear. Aim of the study is to compare MWA with RFA in the treatment of liver cancer.

**Methods:**

Meta-analysis was conducted according to the PRISMA guidelines for studies published from 2010 onwards. A random-effects model was used for the meta-analyses. Complete ablation (CA), local tumor progression (LTP), intrahepatic distant recurrence (IDR), and complications were analyzed.

**Results:**

Four randomized trials and 11 observational studies with a total of 2,169 patients met the inclusion criteria. Although overall analysis showed no significant difference in LTP between MWA and RFA, subgroup analysis including randomized trials for patients with hepatocellular cancer (HCC) demonstrated statistically decreased rates of LTP in favor of MWA (OR, 0.40; 95% CI, 0.18–0.92; *p* = 0.03). No significant differences were found between the two procedures in CA, IDR, complications, and tumor diameter less or larger than 3 cm.

**Conclusions:**

MWA showed promising results and demonstrated better oncological outcomes in terms of LTP compared to RFA in patients with HCC. MWA can be utilized as the ablation method of choice in patients with HCC.

## Introduction

Over the past 30 years, several ablative methods have been developed for the treatment of hepatic cancer as an alternative to surgical resection and liver transplantation in patients with unresectable cancer or in selected patients with resectable disease. Recent guidelines recommend radiofrequency ablation (RFA) and microwave ablation (MWA) as the ablative methods with the highest efficacy in the treatment of liver cancer.^1^, ^2^

Tumor location near to the main biliary tree, abdominal organs, or diaphragm is a relative contraindication for RFA because of the risk of severe complications.^2^ RFA is prone to heat-sink effect, which reduces further the efficacy of the treatment.^1^, ^2^ On the other hand, MWA, which is a more recent thermal ablation technique, is associated with higher intratumoral temperatures, resulting in faster, larger, and more homogenous ablation compared to RFA.^2^ Furthermore, MWA is less prone to heat-sink effect and can be utilized in tumors adjacent to vessels.

According to the European Association for the Study of the Liver (EASL) recommendations, MWA showed promising results for local tumor control in patients with hepatocellular cancer (HCC).^1^ The guidelines by the American Association for the Study of Liver Diseases (AASLD) reported that MWA has potential advantages over RFA; however, further studies are required to provide safety and efficacy data.^2^ The Cochrane meta-analysis conducted in 2013 failed to provide evidence regarding the role of ablative methods in the treatment of HCC since only one randomized clinical trial (RCT) with high risk of bias was available.^3^ The last conducted meta-analysis in 2019 reported beneficial outcomes in favor of MWA.^4^ However, low quality randomized and observational studies, which were affected by confounding bias were included in this meta-analysis, which could influence the reliability of the outcomes.

Despite the promising results of MWA in the treatment of liver cancer, efficacy and safety of MWA compared to RFA is unclear. Aim of this meta-analysis is to compare RFA and MWA in the treatment of HCC and liver metastases. Our hypothesis is that the beneficial characteristics of MWA are translated into better oncological outcomes compared to RFA.

## Methods

### Inclusion and exclusion criteria

A protocol was developed to pre-specify criteria for including and excluding studies in the review. Eligibility criteria were based on the PICO elements (population, interventions, comparators, and outcomes) plus a specification of the type of studies that have addressed these questions. RCTs and observational studies (prospective or retrospective cohort and case-control studies) were eligible for inclusion. Studies conducted before 2010 were excluded from the meta-analysis.

Studies meeting the following criteria were included: (1) population: adults with primary liver cancer or hepatic metastases; (2) interventions: RFA and MWA as monotherapy or combined with surgical resection; (3) MWA and RFA conducted percutaneously, laparoscopically, or through laparotomy; (4) comparators: effectiveness and safety of MWA compared to RFA; (5) outcomes: results provided data relative to complete ablation (CA), local tumor progression (LTP), intrahepatic distant (IDR), complications; (6) full text available in English or German; (7) studies with low or moderate risk of bias.

Exclusion criteria were the following: (1) studies with benign liver tumors; (2) pediatric population; (3) animal or in vitro studies; (4) RFA or MWA combined with other interventions such as transarterial chemoembolization (TACE); (5) gender and geographical criteria were not utilized; (6) stage of liver cancer, size, and location of tumors did not constitute exclusion criteria; (7) duplicate data.

### Study outcomes

Primary outcomes were the CA rates and the LTP. CA was defined as the no enhancement of the tumor in the hepatic arterial or portal venous phase in dynamic enhanced imaging (CT, MRI), which was conducted after ablation. As incomplete ablation was defined the enhancement of the tumor in dynamic enhanced imaging.^5^ As LTP was defined the reappearance of the tumor within or adjacent to the ablation zone during the follow-up period. Studies that reported recurrence rates without to define if that is local or distant were excluded from this analysis. In studies where 1-year, 3-year, and 5-year LTP rates were reported, only the overall 5-year LTP rate was included in the analysis. In the majority of cases, patients were presented with multinodular disease. For that reason, CA and LTP were recorded for every treated lesion. Studies, where LTP and CA were recorded per patient and not per lesion, were excluded from the analysis.

IDR and complications were included in the secondary outcomes. IDR was defined as distant recurrence within the liver. In studies where 1-year, 3-year, and 5-year IDR rates were reported, the overall 5-year rate was included in the meta-analysis. Minor complications, which required no intervention or were not associated with prolonged hospital stay, were not included in the analysis. Major complications were defined as post-interventional events that lead to substantial morbidity or disability, require intervention, and result in prolonged hospital stay.

A subgroup analysis was conducted, comparing CA and LTP for tumors ≤ 3 cm and tumors > 3 cm in diameter. RFA and MWA were compared separately in patients diagnosed with HCC and colorectal liver metastases (CRLM).

### Search strategy and data collection

The systematic review was conducted according to the Preferred Reporting Items for Systematic Reviews and Meta-Analyses (PRISMA) guidelines.^6^ A systematic search of MEDLINE (PubMed and Ovid) and the Cochrane Central Register of Controlled Trials was conducted for relevant systematic reviews, RCTs, and observational studies. Access to Embase was not available for the review team. The search was accomplished in July 2020.

The search strategy included the following keywords: ((((“Carcinoma, Hepatocellular”[Mesh])) OR (hepatic tumor)) AND (“Radiofrequency Ablation”[Mesh])) AND (“Microwaves”[Mesh]). The search strategy was not limited by geographical criteria. English and German language articles were reviewed for inclusion. Studies conducted between 2010 and 2020 were screened. Reference lists of retrieved studies and relevant reviews were hand-searched.

Eligibility for inclusion was evaluated in the title and abstract of each publication. If the title and abstract were relevant to the review question, full-text screening was conducted. Reviewers were not blinded to the name of authors and institutions. Screening of articles was conducted by two reviewers. Discrepancies were resolved by consensus. If consensus was not reached, discrepancies were resolved by adjudication from a third reviewer. Data were extracted independently by two reviewers and checked from a third reviewer. When further information was required during data extraction, the reviewers tried to contact the corresponding author with email.

### Risk of bias assessment

Non-randomized studies were included since available RCTs were limited. The quality of RCTs and observational studies was assessed using the Cochrane Risk of Bias version 2 (RoB 2) tool and The Risk of Bias In Non-randomized Studies of Interventions (ROBINS-I) tool, respectively.^7^ Risk of bias was assessed independently by two reviewers. In case of disagreement, a third author adjudicated the final judgement. High risk RCTs were excluded from the analysis.

Non-randomized studies vary with respect to their intrinsic ability to estimate the causal effect of an intervention. Therefore, to reach reliable conclusions and to eliminate the risk of bias in our results, only studies with low and moderate risk of bias were included in the meta-analysis. Studies with “Serious”, “Critical” risk of bias, or “No information” were excluded from the meta-analysis.

Review authors have defined confounding domains in the review protocol. Confounding domain is a preintervention prognostic factor of the outcome that also predicts whether an individual receives RFA or MWA. Non-randomized studies were assessed as ‘Low Risk of Bias’ in this domain when patients in both groups were matched using propensity score based on the confounding factors. Surveys that compared confounding factors at baseline without propensity score matching and reported no statistical differences were included as studies with ‘Moderate Risk of Bias’. Finally, studies with statistically different baseline characteristics or not reported or not compared baseline characteristics were assessed as ‘Serious Risk’ or ‘Critical Risk’ and were excluded from the analysis.

### Statistical analysis

For all outcomes of interest, meta-analyses for the Odds Ratio (OR) have been performed. The amount of heterogeneity (measured by *I*^2^) among studies varied strongly between outcomes, ranging from very low to substantially. However, in order to be consistent with respect to the modelling strategy, random effects estimates for the OR have been chosen for all outcomes. Sensitivity analyses for this modelling found high agreement between estimates derived from random and fixed effect models. Within the random effects model, the DerSimonian-Laird estimator^8^ has been used for the calculation of between-studies variance (τ^2^) in combination with the Mantel-Haenszel method^9^ for the calculation of between-study heterogeneity statistic Q.^10^ Overall treatment effects (overall ORs) were derived from the random effects models and presented as point estimates and corresponding 95% confidence intervals (CI). In all analyses, *p*-value < 0.05 was regarded as statistically significant.

The amount of heterogeneity among studies has been measured by the *I* value. In addition, tests of heterogeneity were performed on the Q statistic, which provides *p*-values. Funnel plots have been created to examine publication bias in meta-analysis outcomes with more than five included studies. Asymmetry in funnel plots has been analyzed using Egger’s test of the intercept in meta-analysis outcomes with more than ten included studies.^11^ For statistical analysis, the R software for statistical computing (R version 4.0.1, R Core Team, 2020) has been used in combination with the meta package and dmetar package.^12^

## Results

### Studies selection

**Figure 1:**
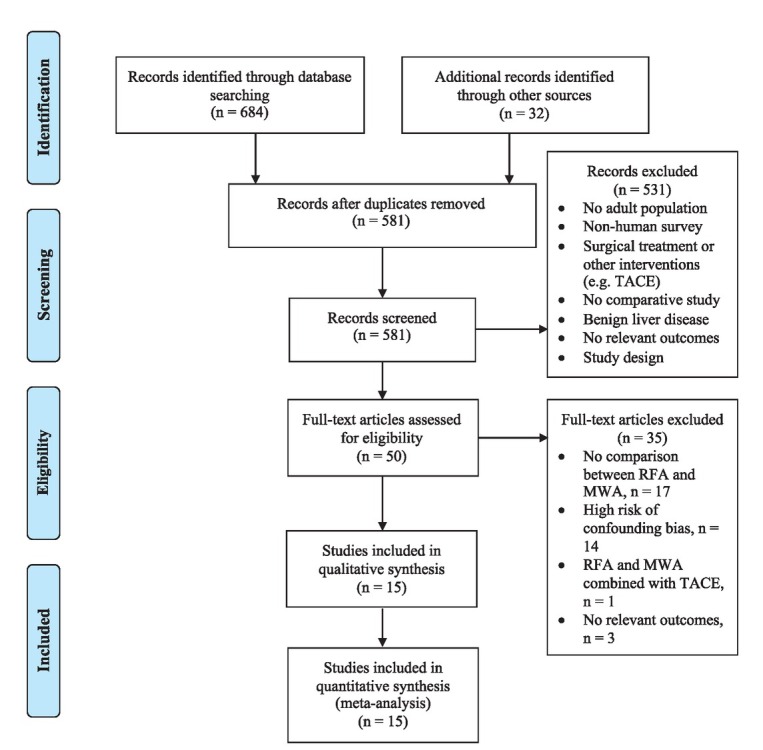
Prisma flow diagram. MWA = microwave ablation, RFA = radiofrequency ablation, TACE = transarterial chemoembolization

A total of 716 publications were identified from database searching. After removing duplicates, 581 unique articles were screened for inclusion. During the title-abstract screening phase, a total of 531 irrelevant studies were excluded. Fifty articles were selected for full-text review. Thirty-five articles were excluded because of no comparison between RFA and MWA (n = 17), increased risk of bias in confounding domain for observation studies (n = 14), combined treatment with TACE (n =1), and no relevant outcomes (n = 3). The RCT by Yu *et al*. was assessed as a trial with high risk of bias and was excluded from the meta-analysis.^13^ Finally, 15 studies (four RCTs, one prospective study, ten retrospective studies), were included in our review. PRISMA diagram is demonstrated in Figure 1.

### Characteristics of included studies

Fifteen studies with a total of 2,169 patients were included in the analysis.^14^, ^15^, ^16^, ^17^, ^18^, ^19^, ^20^, ^21^, ^22^, ^23^, ^24^, ^25^, ^26^, ^27^, ^28^ The recruitment period ranged from 2001 to 2018. The sample size ranged from 40 to 460 patients. The average age across studies ranged from 52 to 68 years. The mean or median tumor size ranged from 1.7 cm to 3.75 cm. All studies reported no significant differences in tumor size between the two treatment groups. Study characteristics and baseline characteristics are demonstrated in Table 1.

**Table 1:** Study and baseline characteristics of studies included in the meta-analysis

Study	County	Study Design	Tumor	RFA, n	MWA, n	Age, RFA	Age, MWA	Child-Pugh A/B/C, RFA	Child-Pugh A/B/C, MWA	Tumor size (cm), RFA	Tumor size (cm), MWA	Tumor lesions, RFA	Tumor lesions, MWA
Kamal 2019 (13)	Egypt	RCT	HCC	28	28	55	55	22.6.2000	22.6.2000	3.28 ± 0.91	3.25 ± 0.92	34	34
Vietti Violi 2018 (14)	France/Switzerland	RCT	HCC	73	71	65 (median)	68 (median)	53/20/0	57/14/0	1.8 ± 0.71	1.8 ± 0.65	104	98
Abdelaziz 2014 (15)	Egypt	RCT	HCC	45	66	56.8 ± 7.3	53.6 ± 5	24/21/0	25/41/0	2.95 ± 1.03	2.9 ± 0.97	52	76
Di Vece 2013 (16)	Italy	RCT	HCC/Metastases	20	20	59 (median)	63 (median)	N/R	N/R	3.2 (median)	3.6 (median)	20	20
Qian 2012 (17)	China	Prospective	HCC	20	22	56 ± 11	52 ± 12	20/0/0	22/0/0	2 ± 0.5	2.1 ± 0.4	20	22
Sparchez 2019 (18)	Romania	Retrospective	Metastases	44	17	60.18 ± 9.96	62.12 ± 10.73	-	-	2.maj	feb.55	62	20
Takahashi 2018 (19)	USA	Retrospective	CRLM	54	51	N/R	N/R	-	-	2.4 (median)	2.1 (median)	155	121
Shady 2018 (20)	USA	Retrospective	CRLM	62	48	N/R	N/R	-	-	1.8 (median)	1.7 (median)	85	60
Xu 2017 (21)	China	Retrospective	HCC	159	301	54 ± 11	54.2 ± 11	140/19/0	278/23/0	1.7 ± 0.3	1.7 ± 0.3	159	301
van Tilborg 2016 (38)	Netherlands	Retrospective	CRLM	Total number of participants: 122		N/R	N/R	-	-	2.apr	2.maj	151	48
Potretzke 2016 (23)	USA	Retrospective	HCC	55	99	62	61	N/R	N/R	2.apr	2.feb	69	136
Zhang X. 2014 (24)	China	Retrospective	HCC/Metastases	92	230	51.5 ± 14.3	55.7 ± 13.2	N/R	N/R	5.4 ± 1.9	5.7 ± 2.1	173	349
Zhang L. 2013 (25)	China	Retrospective	HCC	78	77	54 ± 10.5	54 ± 9.5	78/0/0	77/0/0	2.3 ± 0.4	2.2 ± 0.4	97	105
Liu 2013 (26)	China	Retrospective	Metastases	54	35	53.1 ± 12.7	53.4 ± 15.3	-	-	2.5 ± 1.0	2.3 ± 1.0	70	62
Ding 2013 (27)	China	Retrospective	HCC	85	113	58.64 ± 8.52	59.06 ± 11.68	49/36/0	75/30/0	2.38 ± 0.81	2.55 ± 0.89	98	131

Age and tumor size are recorded as mean, mean ± standard deviation (SD), or median. CRLM = colorectal liver metastases, HCC = hepatocellular cancer, MWA = microwave ablation, RCT = randomized clinical trial, RFA = radiofrequency ablation, N/R = not reported

Eight studies evaluated the role of thermal ablation in patients with HCC.^14^, ^15^, ^16^,^18^,^22^,^24^,^26^,^28^ Child-Pugh score, which was estimated in the majority of studies, was not statistically different between RFA and MWA groups. In the retrospective study conducted by Potretzke *et al*., MELD score was estimated, which was similar in the RFA and MWA group.^24^ Four studies included patients with hepatic metastases of different origins^17^,^19^,^25^,^27^, whereas three studies included only patients with CRLM.^20^,^21^,^23^ In the RCT conducted by Di Vece *et al*., the primary origin of liver metastases was not reported.^17^

### Quality assessment

The quality of included RCTs was acceptable (Supporting Information, Figure S1). Two out of four RCTs were judged to be at low risk of bias across all domains.^15^,^17^ The RCT conducted by Abdelaziz *et al*. was judged to raise some concerns in bias due to deviations from intended interventions since important non-protocol interventions during follow-up were not recorded.^16^

Three studies reported the method of randomization and allocation sequence generation. Coin flip^16^ and centralized computer-generated randomization^15^,^17^ were utilized as methods for random sequence generation. In these RCTs, the allocation sequence was adequately concealed. The study by Kamal *et al*. did not report the method of randomization and was judged to raise some concerns in the domain of bias arising from the randomization process.^14^ Simple randomization was used in two studies^4^,^16^, whereas the other two RCTs utilized blocked-restricted randomization.^15^,^17^

Physicians, who conducted the ablations, were not blinded, since different equipment was utilized in each treatment modality. Patients were masked to the treatment in one trial.^15^ In two RCTs, independent outcome assessors, who were masked to the treatment allocation, reviewed all images and recorded the outcomes.^15^,^17^ In the studies conducted by Kamal *et al*. and Abdelaziz *et al*., outcome assessors were not blinded.^14^,^16^ However, the risk of bias due to blinding of outcome assessors was considered to be low, since assessment of CT or MRI imaging was objective and specific criteria were utilized for the evaluation of CA and LTP.

All retrospective studies were judged to be at moderate risk of overall and confounding bias (Supporting Information, Table S1). Studies that evaluated the role of ablation in hepatic metastases did not report the histological stage of the primary tumors.^19^, ^20^, ^21^,^23^,^25^, ^27^ Two studies that included HCC patients did not compare the BCLC stage at baseline.^25^,^26^ Tumor size was comparable between the two groups in all studies.

Four studies were affected by selection bias.^18^,^20^,^22^, ^23^ In these studies, the number of excluded patients and the reason of exclusion were not reported. Bias due to deviations from intended interventions was seen only in the survey by van Tilborg *et al*.^23^ Eleven patients underwent retreatments during follow-up, using the alternative ablation technique.

### Meta-analysis outcomes

#### Complete ablation

**Figure 2:**
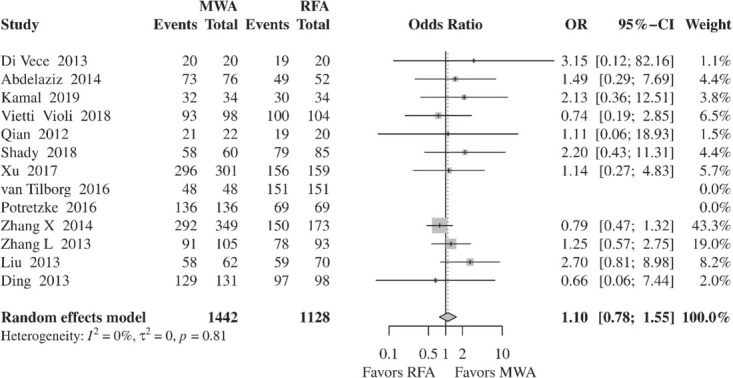
Forest plot of random-effects meta-analysis results for complete ablation rates in the MWA and RFA group. CI = confidence interval, MWA = microwave ablation, OR = odds ratio, RFA = radiofrequency ablation

Non-significant difference was found in CA rates between MWA and RFA (OR, 1.10; 95% CI, 0.78– 1.55; *p* = 0.5898) (Figure 2). No evidence of heterogeneity was found between the included studies (*I*^2^, 0%; τ^2^, 0%, *p* = 0.81). In order to evaluate the influence of retrospective studies in the results, a further analysis was performed, calculating OR separately for RCTs and retrospective studies (Supporting Information, Figure S2). Since only one prospective study was included in the meta-analysis^18^, further stratification by prospective studies was not performed. For the four RCTs, meta-analysis outcomes remained consistent with the main overall results (OR, 1.28; CI, 0.54–3.05; *p* = 0.5706). Similarly, meta-analysis of the retrospective studies showed no significant difference between the two approaches (OR, 1.07; CI, 0.73–1.56; *p* = 0.7373).

#### Local tumor progression

**Figure 3:**
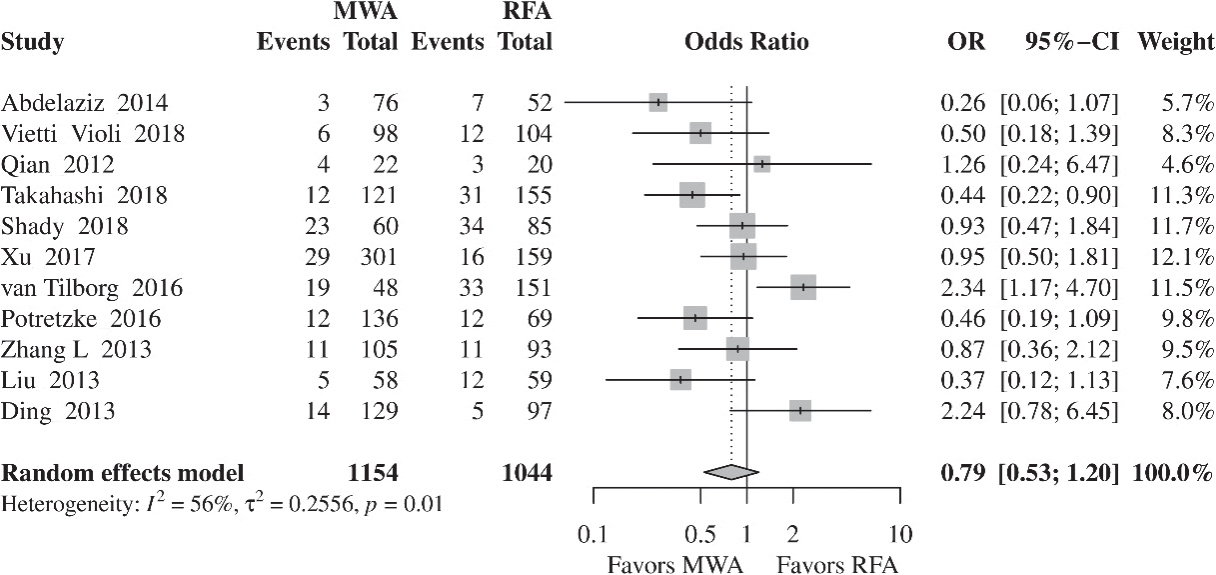
Forest plot of random-effects meta-analysis results for local tumor progression in the RFA and MWA group. CI = confidence interval, MWA = microwave ablation, OR = odds ratio, RFA = radiofrequency ablation

**Figure 4:**
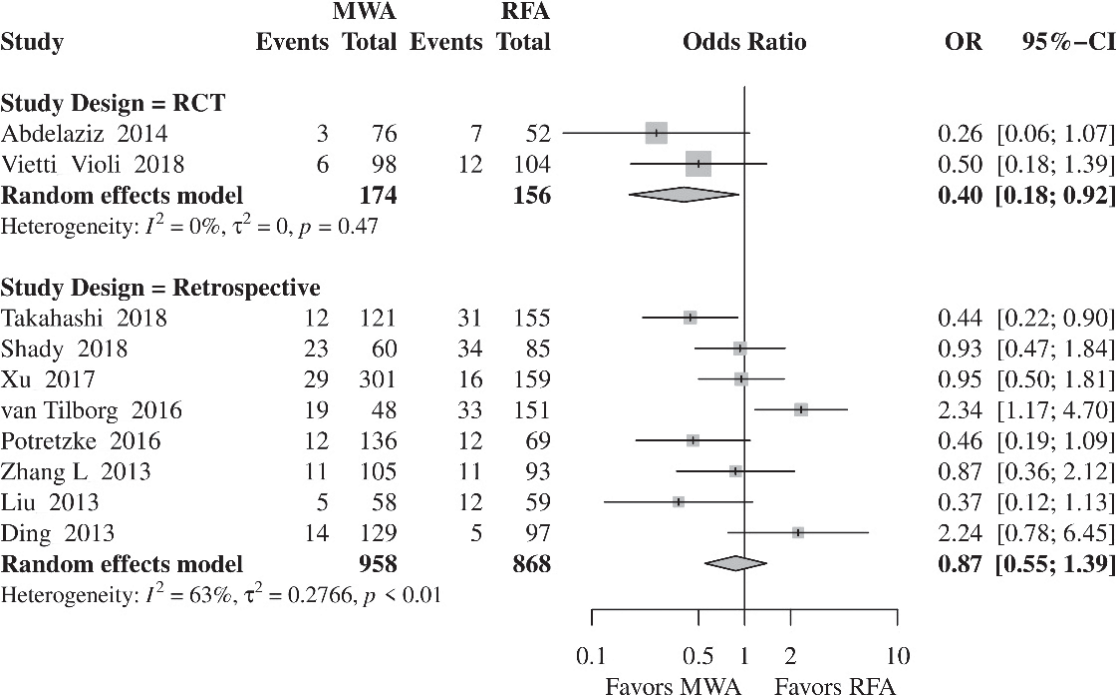
Forest plot of random-effects meta-analysis results for local tumor progression in the RFA and MWA group based on the study type. CI = confidence interval, MWA = microwave ablation, OR = odds ratio, RFA = radiofrequency ablation

LTP rates were comparable between MWA and RFA (OR, 0.79; 95% CI, 0.53–1.20; *p* = 0.2689) (Figure 3). However, inter-study heterogeneity was significant (*I*^2^, 56%; τ^2^, 0.2556; *p* = 0.01). In the subgroup analysis, which included two RCTs, significantly reduced rates of LTP were found in the MWA group compared to RFA (OR, 0.40; 95% CI, 0.18–0.92; *p* = 0.03). Furthermore, inter-study heterogeneity was not significant (*I*^2^, 0%; τ^2^, 0; *p* = 0.47). On the other hand, in the subgroup analysis of retrospective studies, the rates of LTP were similar in both groups (OR, 0.87; 95% CI, 0.55–1.39; *p* = 0.5731), whereas heterogeneity remained significant (*I*^2^, 63%; τ^2^, 0.2766; *p* < 0.01) (Figure 4).

#### Intrahepatic distant recurrence

**Figure 5:**
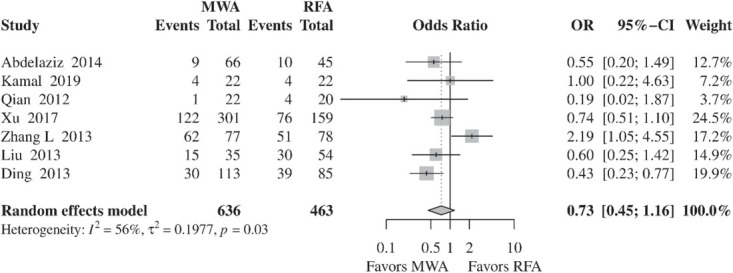
Forest plot of random-effects meta-analysis results for intrahepatic distant recurrence rates in the RFA and MWA group. CI = confidence interval, MWA = microwave ablation, OR = odds ratio, RFA = radiofrequency ablation

Analysis of seven studies showed no statistically significant differences in IDR between MWA and RFA (OR, 0.73; 95% CI, 0.45–1.16; *p* = 0.1826) (Figure 5). Inter-study heterogeneity was significant (*I*^2^, 56%; τ^2^, 0.1977; *p* = 0.03). Meta-analysis of RCTs showed no significant difference between the two procedures (OR, 0.66; 95% CI, 0.29–1.52; *p* = 0.3266). No evidence of heterogeneity was found between the two randomized trials (*I*^2^, 0%; τ^2^, 0; *p* = 0.52). Similarly, no difference between RFA and MWA was demonstrated when only retrospective studies were included in the meta-analysis (OR, 0.79; 95% CI, 0.43–1.46; *p* = 0.4529). However, heterogeneity among retrospective studies was statistically significant (*I*^2^, 75%; τ^2^, 0.2848; *p* < 0.01).

#### Complications

**Figure 6:**
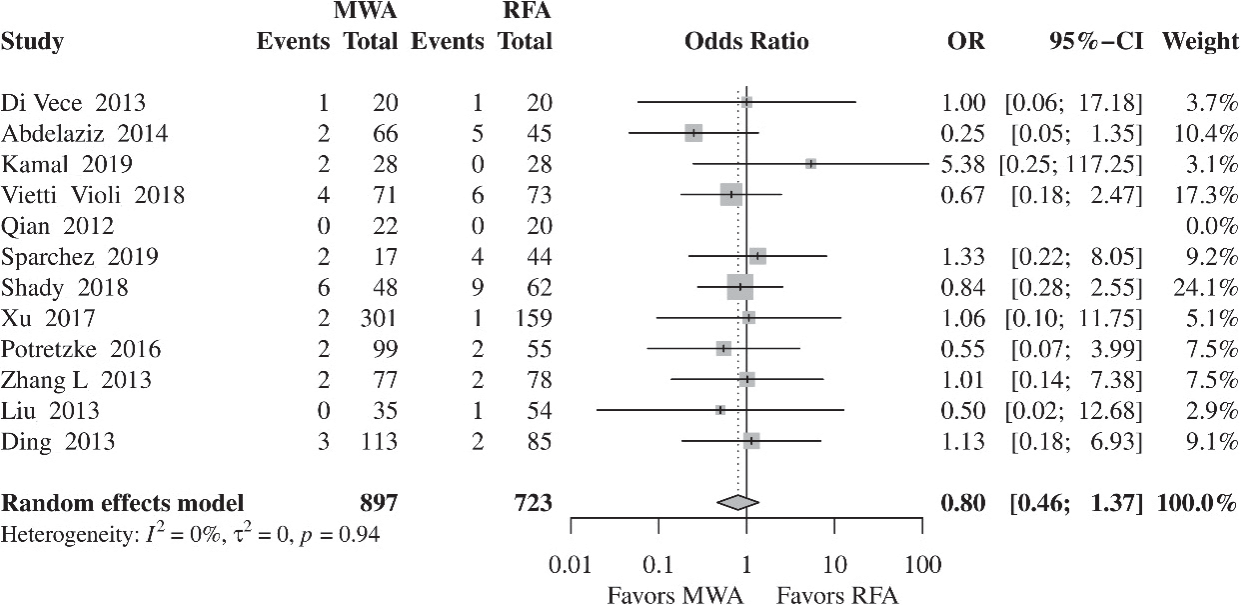
Forest plot of random-effects meta-analysis results for complication rates following RFA and MWA. CI = confidence interval, MWA = microwave ablation, OR = odds ratio, RFA = radiofrequency ablation

The most commonly reported major complications in both groups were subcapsular hepatic hematoma, perihepatic hematoma, arterial bleeding requiring embolization or surgical treatment, hepatic abscess, biliary fistula, bowel perforation, abdominal wall skin burn, and pleural effusion. The risk of major complications was not different between the two approaches (OR, 0.80; 95% CI, 0.46–1.37; *p* = 0.4129) (Figure 6). In the subgroup meta-analysis, comparing RFA and MWA based on the type of study, results remained consistent without significant differences in the rate of complications in the RCTs^14^, ^15^, ^16^, ^17^ and retrospective studies.^19^,^21^, ^22^,^24^,^26^, ^27^, ^28^,

#### Tumor size

Four studies assessed the rates of CA in patients with tumor < 3 cm.^6^,^18^,^24^,^28^ Heterogeneity among the surveys was not significant (*I*^2^, 0%; τ^2^, 0; *p* = 0.54). Results of meta-analysis showed no significant difference in CA between RFA and MWA (OR, 2.18; 95% CI, 0.34–13.88; *p* = 0.4095). For the outcome of LTP, three studies were included in the meta-analysis.^18^,^24^,^28^ Results revealed no significant differences between the two modalities (OR, 0.99; 95% CI, 0.49–2.01, *p* = 0.9729).

Regarding tumors with size larger than 3 cm, three studies reported CA rates^16^,^24^,^28^ and two studies evaluated LTP.^24^,^28^ Meta-analysis showed no significant difference in CA and LTP between RFA and MWA (*p* = 0.7682; *p* = 0.8168, respectively).

#### Hepatocellular cancer

Meta-analysis showed no significant difference in CA between RFA and MWA in patients with HCC (OR, 1.18; 95% CI, 0.70–1.99; *p* = 0.5437). When only RCTs were included in the meta-analysis^14^, ^15^, ^16^, , the results remained constant and significant differences were not found (OR, 1.20; 95% CI, 0.49–2.94; *p* = 0.6904).

LTP was not significantly different between RFA and MWA (OR, 0.77; 95% CI, 0.49–1.22, *p* = 0.2723). However, when only pooling RCTs^15^,^16^ rates of LTP were statistically decreased in the MWA group compared to RFA (OR, 0.40; 95% CI, 0.18–0.92, *p* = 0.03). On the other hand, meta-analysis results of the retrospective studies^22^,^24^,^26^,^28^ showed no difference between the two procedures (OR, 0.92; 95% CI, 0.52–1.60; *p* = 0.7614).

Differences between RFA and MWA in the incidence of IDR were not found (OR, 0.75; 95% CI, 0.43–1.30; *p* = 0.3041). However, heterogeneity among surveys was significant (*I*^2^, 63%; τ^2^, 0.2594; *p* = 0.02). Subgroup analysis of RCTs^14^,^16^ and retrospective studies^22^,^26^,^28^ showed no statistically different results between the two procedures (*p* = 0.3266; *p* = 0.6975, respectively). Inter-study heterogeneity was not significant across RCTs; however, heterogeneity remained significant among retrospective studies.

#### Colorectal liver metastases

CA and LTP were compared between RFA and MWA in patients with CRLM. Meta-analysis included three retrospective studies.^20^,^21^,^23^ For both outcomes, no significant differences were found between the two procedures (*p* = 0.3441; *p* = 0.9826, respectively).

#### Publication bias

CA, LTP, IDR, complications, CA in HCC patients, LTP in HCC patients, and IDR in HCC patients were examined for publication bias (Supporting Information, Figure S3, S4). Results demonstrated a low risk of publication bias for the outcomes assessed. Egger’s test was utilized in the outcomes with more than ten included studies. No obvious asymmetry or *p*-value < 0.05 were detected, which is associated with no evidence of publication bias.

## Discussion

RFA is currently one of the most widely used thermal ablation modalities. On the other hand, utilization of MWA has been increased the last years as a result of significant advancements in technology of new generation devices. These advancements are translated into higher temperatures and faster heating compared to RFA, large ablation volumes, and less heat sink effect.^29^ However, MWA has not been adequately compared with RFA and selection of appropriate treatment is not based on high level of evidence.^30^ On the basis of these considerations, we conducted the present meta-analysis to evaluate the role of MWA in the treatment of liver cancer.

Meta-analysis of CA rates, which included more than 2,500 tumor lesions, demonstrated no significant differences between MWA and RFA. In the subgroup analysis of RCTs with 438 tumors, similar rates of CA were found between the two methods. Analysis of all included studies revealed no significant difference in LTP between MWA and

RFA. Since increased heterogeneity was detected among the studies, subgroup analysis of RCTs was conducted to decrease heterogeneity and to evaluate the influence of observational studies on the outcomes. The RCTs by Abdelaziz *et al*. and Vietti Violi *et al*. included 255 patients with HCC and up to three lesions with less than 5 cm and 4 cm tumor size, respectively.^15^,^16^ Furthermore, new generation MWA devices with 2,450 MHz generators were utilized. Meta-analysis of the two RCTs demonstrated statistically decreased rates of LTP in the MWA group. Specifically, LTP was reported in 5.2% and 12.2% of tumor lesions treated with MWA and RFA, respectively.

The finding of the RCTs is consistent with the physics and characteristics of radiofrequency and microwave energies. MWA is associated with higher temperatures, faster heating, larger ablation volumes, and less heat sink effect compared to RFA, which are translated into better oncological outcomes in terms of LTP in the present meta-analysis. On the other hand, meta-analysis of retrospective studies failed to demonstrate superiority of MWA over RFA, which is attributed to the significant inter-study heterogeneity.

Consequently, though CA was comparable between the two procedures, LTP was beneficial in favor of MWA. These conflicting results are not surprising given the limitations associated with measurement and evaluation of complete ablation response. Imaging modalities cannot detect with 100% accuracy whether neoplastic cells have been sufficiently ablated. For that reason, ablation response cannot be considered as the most reliable indicator of treatment effectiveness. On the other hand, follow-up imaging examinations and LTP have been considered of great importance in detecting treatment failure. LTP is the most reliable indicator of treatment effectiveness and can be utilized as assessment tool of treatment efficacy.

IDR was comparable between the two ablative methods. Subgroup analysis of two RCTs demonstrated similar rates of IDR between MWA and RFA. The RCT by Kamal *et al*. reported IDR rates of 18.2% at 12-month follow-up^14^, while the survey by Abdelaziz *et al*. reported rates between 13.6% and 22.22% at 27-month follow-up.^16^ The beneficial outcomes in LTP were not associated with a decreased incidence of intrahepatic recurrence in the MWA group. This result is attributed to a variety of factors, which are associated with cancer disease, underlying liver disease, and indications of treatment. Patients were oft assigned to treatment based on tumor proximity to blood vessels or biliary tract. These tumors are characterized by increased incidence of local metastases, which in the majority of cases cannot be prevented with an effective ablation therapy. Furthermore, an underlying hepatic disease in patients with HCC or an advanced primary tumor in patients with hepatic metastases are predisposing factors for tumor recurrence, which cannot be eliminated with an ablation procedure.

The risk of complications was not significantly different between the groups and both procedures presented a limited number of adverse events. This finding is important since larger ablation zones, which are achieved through MWA, could be perceived to cause more perioperative complications and damage to liver function compared to RFA. This assumption was refuted with the results of our meta-analysis.

CA and LTP were compared separately among patients with HCC and CRLM. As mentioned above, results derived from the two RCTs in HCC patients showed statistically decreased rates of LTP following MWA compared to RFA.^15^, ^16^ In the present meta-analysis, only three retrospective studies compared the two methods in patients with CRLM; consequently, reliable conclusions cannot be drawn, though results showed no significant difference.

In accordance with our results, previous studies reported similar rates of CA between RFA and MWA.^4^,^31^, ^32^, ^33^, ^34^ Glassberg *et al*. reported statistically decreased rates of LTP in the MWA group compared to RFA. Systematic reviews and meta-analyses conducted before 2015 reported comparable rates of LTP between RFA and MWA.^31^, ^32^, ^33^, ^34^ However, results were derived from studies that in many cases utilized first generation MWA devices. In our meta-analysis, studies published before 2010 were excluded to eliminate this factor. Since the majority of surveys in our analysis utilized new generation devices, which provide controlled and enhanced ablation, beneficial results of MWA over RFA can be attributed to this factor.

Subgroup analysis showed no difference between RFA and MWA for tumor size less or larger than 3 cm. Similar to our findings, Luo *et al*. concluded that CA and LTP were comparable between RFA and MWA in tumors with diameter larger than 3 cm.^34^ In contrast to our results, Facciorusso *et al*. reported significantly decreased incidence of LTP in the MWA group compared to RFA when meta-analysis was restricted to studies with high tumor burden.^32^ However, the authors failed to define the size of lesions with high tumor burden. This subgroup analysis was performed without clear criteria and results should be evaluated with caution.

In contrast to our results, Glassberg *et al*. found that LTP in patients with tumor sizes > 2.5 cm was statistically reduced in MWA group compared to RFA.^4^ However, authors did not report the studies that were included in this subgroup analysis. For that reason, level and quality of evidence cannot be assessed. At this point, we should mention that Glassberg *et al*. included observational studies with low quality, which were excluded from our meta-analysis, since were associated with high risk of confounding bias and insufficient comparison of baseline characteristics.^35^, ^36^, ^37^, ^38^, ^39^, ^40^ Furthermore, studies that compared RFA or MWA combined with TACE were included in the meta-analysis by Glassberg *et al*., which could influence the results of the ablation methods.

Contrary to our findings, the meta-analysis conducted by Glassberg *et al*. reported that distant recurrence was significantly reduced by 15% with MWA compared to RFA when only RCTs were included in the subgroup meta-analysis.^4^ These results were derived from the RCTs conducted by Abdelaziz *et al*. as well as by Yu *et al*.^13^,^16^ The second RCT was assessed as high risk of bias in all domains during full-text screening in our study. Consequently, results from a high risk study cannot be assessed as reliable and interpretation should be performed with caution.

The findings in the present meta-analysis should be interpreted in view of certain limitations. First, observational studies without randomization were included in the analysis, which is associated with potential confounding, selection, measurement, and reporting bias. In order to eliminate bias attributed to observational studies, only surveys with low or moderate overall risk of bias were included. Second, significant inter-study heterogeneity was observed for certain outcomes. In these cases, influence of retrospective studies on the results and sources of heterogeneity were examined with subgroup analysis of RCTs and retrospective studies separately. Third, different MWA and RFA devices were utilized across the surveys, which could influence the results of our analysis. Since various devices were used, a subgroup analysis based on the type of devices was not possible. Fourth, limited number of studies included patients with liver metastases or CRLM. Consequently, further RCTs are required to compare MWA with RFA in patients with hepatic metastases.

In addition, in the present study, the proved superiority of MWA over RFA in terms of LTP cannot be translated into better long-term oncological outcomes, since survival outcomes were not evaluated. Overall survival and disease-free survival were not included in our analysis, since limited data can be drawn from the available studies. The majority of surveys were retrospective in design and have included patients with no 100% matching in oncological characteristics. Furthermore, some patients underwent simultaneously surgical resection and ablation. Survival of these patients is multifactor in etiology and causality. Regarding patients with liver metastases, neoadjuvant or adjuvant treatment and tumor stage were not 100% similar between the two groups. For that reason, survival after ablation is associated with several parameters, which could not be attributed only to the effectiveness of the ablative procedures. In fact, LTP and CA are generally considered the best indicators of treatment effectiveness for ablative methods rather than overall survival or disease-free survival.

The meta-analysis is strengthened by its broad inclusion of 15 studies with a total of 2,169 patients. In contrast to other meta-analyses, low quality studies were excluded. Consequently, results were derived from high or moderate quality studies. Taking into consideration the results of the present meta-analysis, we suggest that MWA should be the ablation method of choice in the treatment of HCC. Finally, since the majority of studies included patients with HCC, further RCTs are required to evaluate the role of ablation treatments in patients with liver metastases.

## Supporting Information

Microwave ablation compared with radiofrequency ablation for the treatment of liver cancer: a systematic review and meta-analysis
