# Supplementary material for: Microwave Ablation Compared with Radiofrequency Ablation for The Treatment of Liver Cancer: a Systematic Review and Meta-analysis
Source: Radiol Oncol. 2021 Jun 25;55(3):247–58. doi: 10.2478/raon-2021-0030 (PMC8366737; doi:10.2478/raon-2021-0030)
Supplement: Supplementary file 1 — Microwave ablation compared with radiofrequency ablation for the treatment of liver cancer: a systematic review and meta-analysis [file raon-55-247_SM.pdf]

Radiology and Oncology | Ljubljana | Slovenia | www.radioloncol.com

Microwave ablation compared with radiofrequency ablation for the treatment of liver cancer: a systematic review and meta-analysis

Antonios E. Spiliotis, Gereon Gäbelein, Sebastian Holländer, Philipp-Robert Scherber, Matthias Glanemann, Bijendra Patel

doi: 10.2478/raon-2021-0030

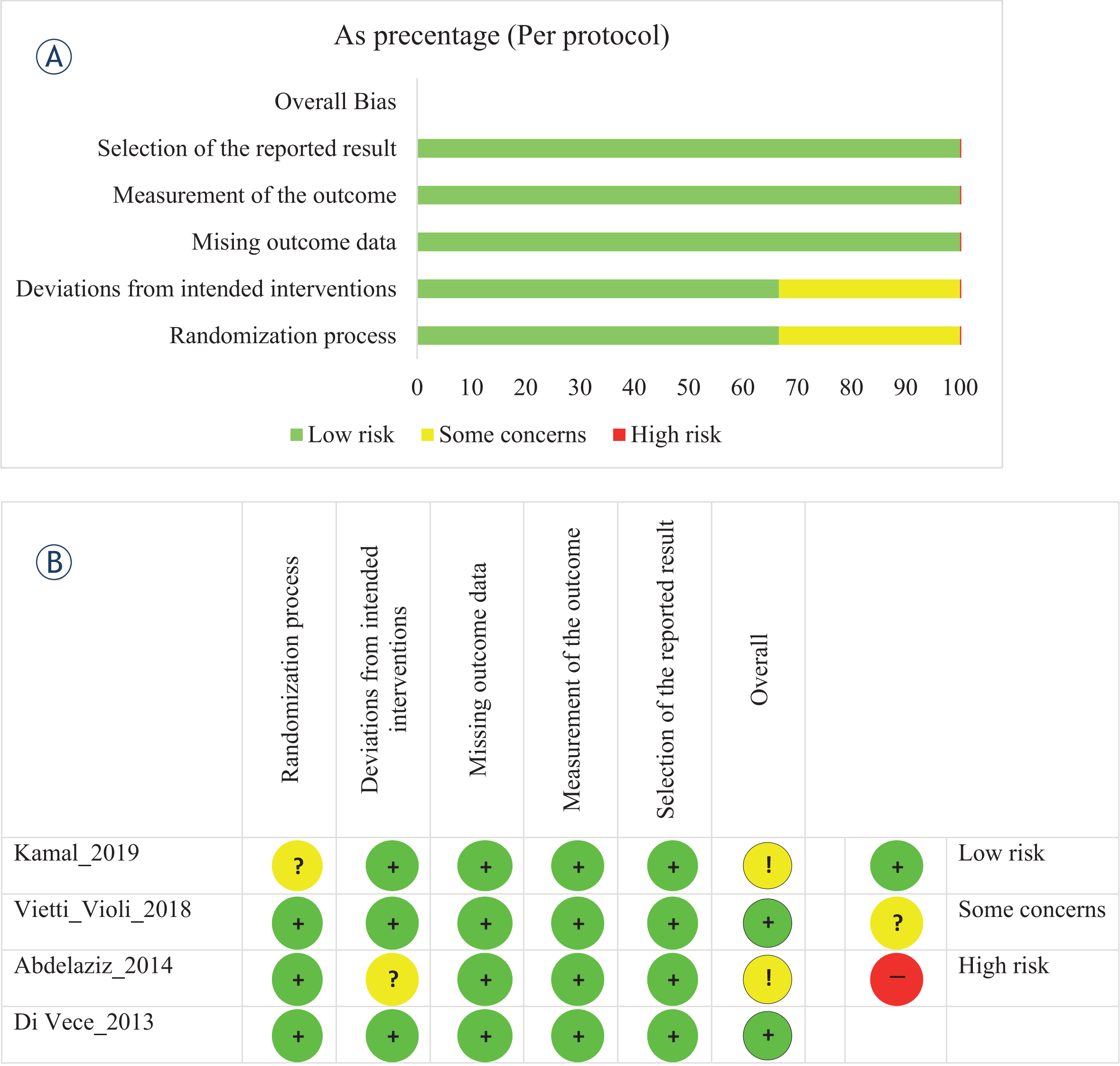

FIGURE S1. Methodological quality assessment of RCTs using the Cochrane RoB tool: **(A)** risk of bias graph, **(B)** risk of bias summary.

RCTs = randomized clinical trials

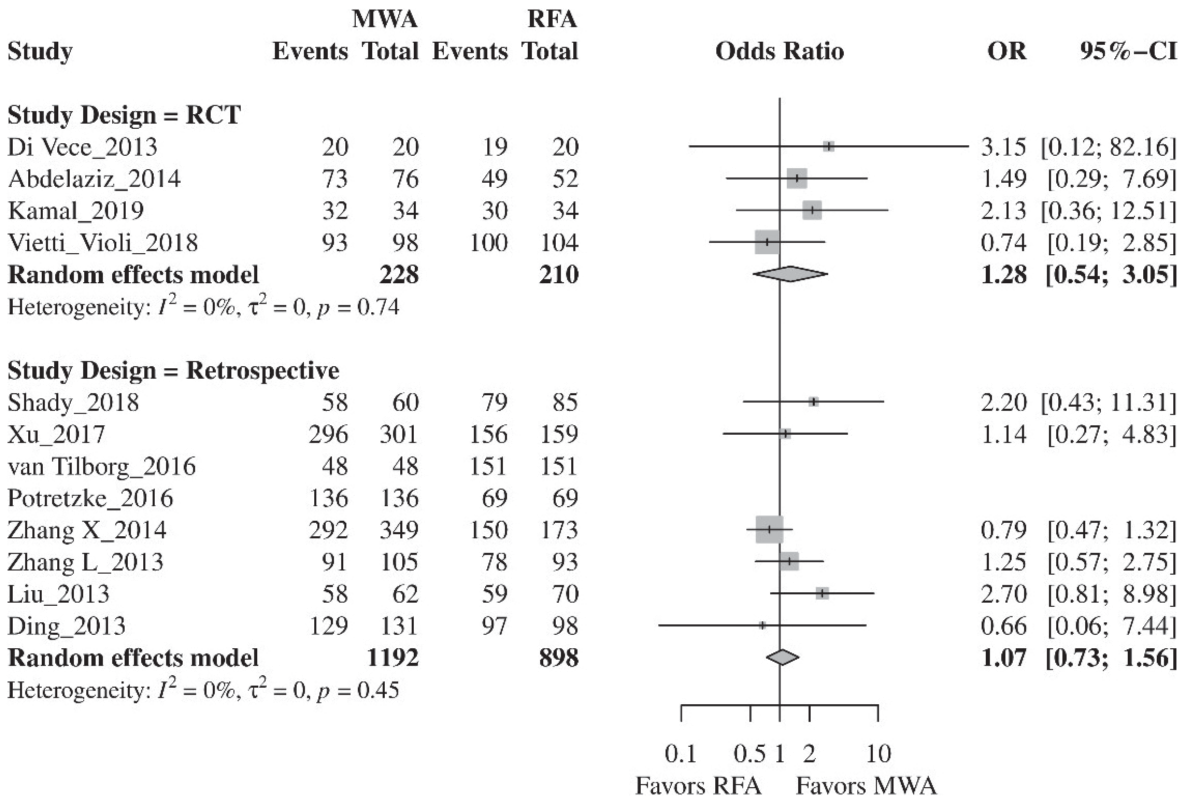

FIGURE S2. Forest plot of random-effects meta-analysis results for complete ablation rates in the RFA and MWA group, stratified by RCTs and retrospective studies.

CI = confidence interval, MWA = microwave ablation, OR = odds ratio, RCTs = randomized clinical trials, RFA = radiofrequency ablation

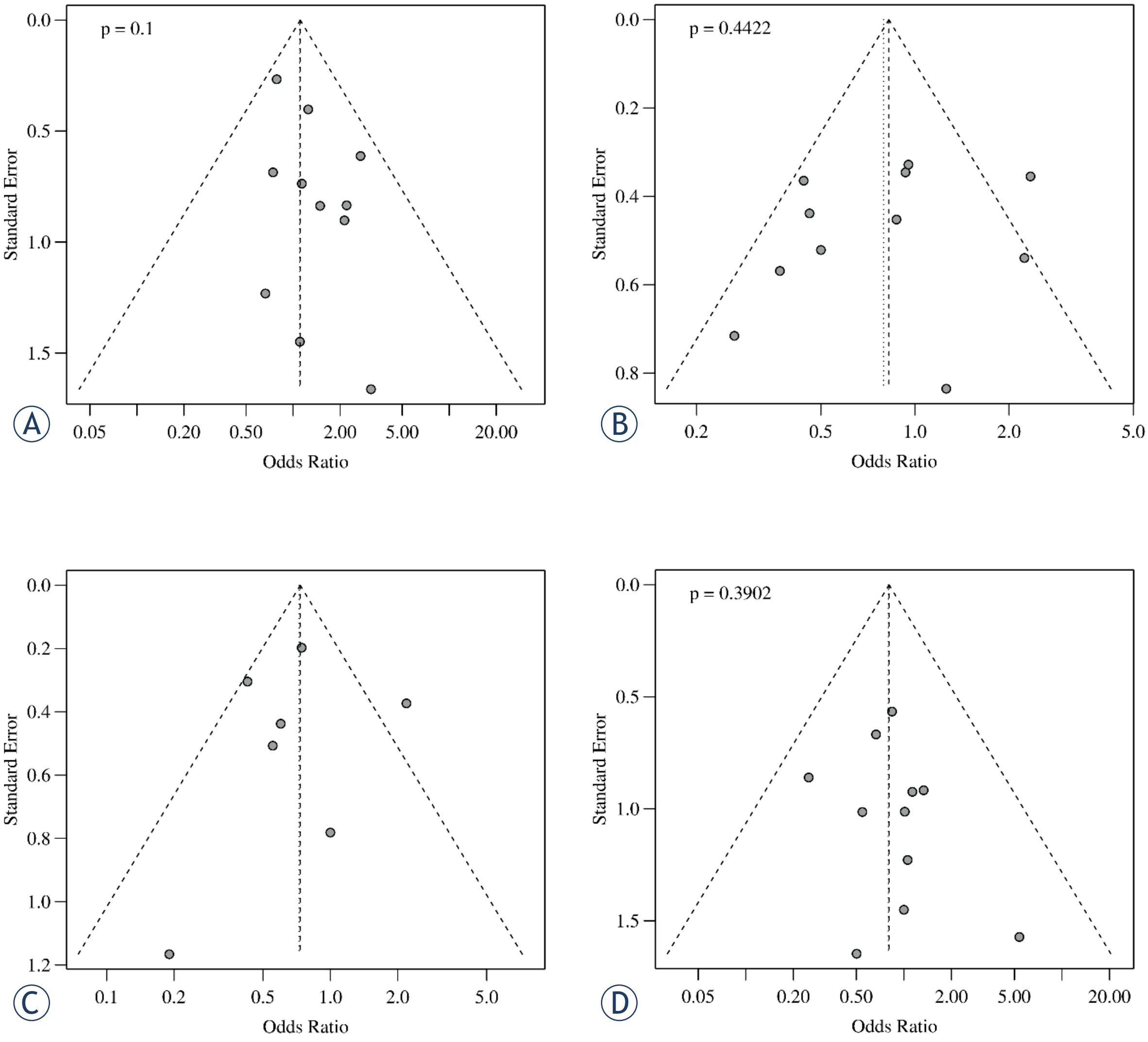

FIGURE S3. Funnel plots assessing publications bias for **(A)** CA, **(B)** LTP, **(C)** IDR, and **(D)** complications. Publication bias was not significant in all outcomes.

CA = complete ablation, IDR = intrahepatic distant recurrence, LTP = local tumor progression

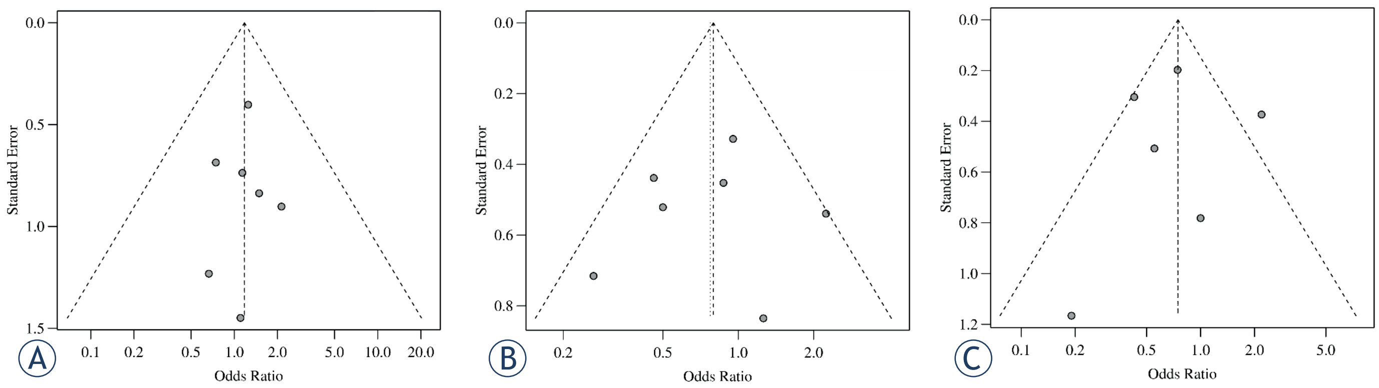

FIGURE S4. Funnel plots assessing publications bias for **(A)** CA, **(B)** LTP, and **(C)** IDR in studies that included HCC patients. Publication bias was low in all outcomes.

CA = complete ablation, IDR = intrahepatic distant recurrence, HCC = hepatocellular cancer, LTP = local tumor progression

TABLE S1. Methodological quality assessment for non-randomized trials using the Cochrane ROBINS-I tool

Study	Confounding bias	Selection bias	Classification bias	Deviation bias	Missing data	Measurement bias	Reporting bias	Total bias
Qian 2012	Low risk	Moderate risk	Low risk	Low risk	Low risk	Low risk	Low risk	Moderate risk
Sparchez 2019	Moderate risk	Low risk	Low risk	Low risk	Low risk	Low risk	Low risk	Moderate risk
Takahashi 2018	Moderate risk	No information	Low risk	Low risk	Low risk	Low risk	Low risk	Moderate risk
Shady 2018	Moderate risk	Low risk	Low risk	Low risk	Low risk	Low risk	Low risk	Moderate risk
Xu 2017	Moderate risk	Moderate risk	Low risk	Low risk	Low risk	Low risk	Low risk	Moderate risk
van Tilborg 2016	Moderate risk	Moderate risk	Low risk	Moderate risk	Low risk	Low risk	Low risk	Moderate risk
Potretzke 2016	Moderate risk	Low risk	Low risk	Low risk	Low risk	Low risk	Low risk	Moderate risk
Zhang X 2014	Moderate risk	Low risk	Low risk	Low risk	Low risk	Low risk	Low risk	Moderate risk
Zhang L 2013	Moderate risk	Low risk	Low risk	Low risk	Low risk	Low risk	Low risk	Moderate risk
Liu 2013	Moderate risk	Low risk	Low risk	Low risk	Low risk	Low risk	Low risk	Moderate risk
Ding 2013	Moderate risk	Low risk	Low risk	Low risk	Low risk	Low risk	Low risk	Moderate risk
